# Direct Detection and Quantification of Aqueous Proteins via a Fluorescent Probe Through the Use of Fluorophore-Induced Plasmonic Current

**DOI:** 10.3390/bios15030150

**Published:** 2025-02-27

**Authors:** Daniel R. Pierce, Chris D. Geddes

**Affiliations:** Department of Chemistry and Biochemistry, Institute of Fluorescence, University of Maryland, Baltimore County, 701 E Pratt St, Baltimore, MD 21202, USA; dan38@umbc.edu

**Keywords:** fluorophore-induced plasmonic current, metal-enhanced fluorescence, surface-enhanced fluorescence, protein sensing, protein quantification

## Abstract

We report on the recent advancements in the sensing of proteins, both directly and with the use of a fluorescent probe, through the use of Fluorophore-Induced Plasmonic Current (FIPC). FIPC are a phenomenon where a fluorophore or excited state species is in close proximity to a plasmonically active metal nanoparticle film (MNF), and the excited state is able to couple to the particle, ultimately leading to enhanced spectroscopic properties. This phenomenon is similar to the well-reported metal-enhanced fluorescence (MEF) phenomenon, wherein the coupled complex produces an enhanced fluorescence emission and a shorter lifetime. However, if the particles themselves are sufficiently spaced and oriented, an induced current can transfer from each discreet particle to the next, creating a detectable current across the film. This detectable current has a magnitude that is proportional to the fluorescent properties of the species that produced it, and can be affected by the polarization of the excitation source; the spacing and size of the particles on the film; the overlap between the spectral properties of the film and the species; as well as externally applied voltages and currents. In this study, we examined whether it is possible to detect protein species, directly due to both their intrinsic fluorescent and absorptive properties, and how that compares to commercially available protein detection probes, in a similar manner to prior work by our group addressing analyte detection via *turn-on* fluorescent probes. This FIPC-based detection technique is a novel method that has not been used for the detection of proteins, and the use of this method could expand the dynamic sensing range of first-pass testing, while overcoming some of the physical limitations on the upper limit of detection of both absorption spectroscopy and fluorescence emission spectroscopy. Our experiments sought to highlight the selectivity of FIPC-based detection relative to both fluorescence and absorption spectroscopy, as well as its sensitivity when working with protein analytes. We examined the effects of protein concentration, intrinsic fluorescent properties, and *turn-on* probes, as well as how these techniques compare to traditional analytical techniques used today.

## 1. Introduction

To understand how Fluorophore-Induced Plasmonic Current (FIPC)-based detection works, it is important to understand the underlying mechanism of Fluorophore-Induced Plasmonic Current (FIPC). This phenomenon is closely tied to the well-reported and studied metal-enhanced fluorescence (MEF) technology, sometimes also called surface-enhanced fluorescence or plasmon-enhanced fluorescence [1,2,3,4,5,6,7,8,9,10,11,12,13,14]. In MEF, an excited state fluorophore will emit its typical fluorescent quanta, until it is brought into close proximity (~10 nm) to a plasmonically active nanoparticle, where, if there is a strong spectral overlap between the particle and the fluorophore, they are able to electromagnetically couple. This new fluorophore–nanoparticle complex subsequently produces a new type of fluorescence emission, one that is significantly brighter and has a substantially reduced excited-state lifetime. It is believed that the nanoparticle in the complex functions as an antenna to initially draw more light into the system, while also providing a more energetically faster and favorable route for emission. This combined system has been of great interest to researchers as it allows for a significantly higher fluorescent intensity relative to the amount of probe used, leading to either significantly reduced material costs or an enhanced limit of detection in fluorescence-based assays. Research by our group on the electrical relationship between MEF and the nanoparticle films they draw their enhancement from has shown that if one applies a small current across the surface of the film, you can selectively modulate whether or not the MEF is enhanced [15,16]. This finding encouraged our research into the electrical relationship between the coupling of these excited-state compounds and their ability to transfer energy between themselves.

The research on Fluorophore-Induced Plasmonic Current (FIPC) is an extension of MEF and plasmonic film coupling research, and focuses on the electrical stimulus of the system upon excitation of the fluorophore [17,18,19,20,21,22,23]. Previous work has shown that if the nanoparticles within the nanoparticle films that are used for MEF enhancement are spaced further apart (such that the individual particles are isolated, described as “island-like”), they are able to take up a portion of the energy from the excited-state coupling and charge, similar to a capacitor [17,18]. Once these nanoparticles are fully charged, they then discharge this energy in the form of electron hopping from one isolated particle to the next, and this transfer of electrons can be detected as a current across the entire surface of the film. This current directly results after excitation and rapidly decays to zero once the fluorophore excitation source is removed. The magnitude of this current response is proportional to the fluorophores’ excited-state characteristics, i.e., their quantum yield, polarizability, molar extinction coefficient at a given wavelength, and ultimately, their concentration [17,18,19,20,21,22,23]. An example experimental schematic for this type of analysis is provided in Figure S1.

In this study, we investigated the capacity of FIPC technology to directly detect proteins in aqueous solutions via intrinsic fluorescence and light absorption at 280 nm, the fluorescent properties of green fluorescent protein (GFP), and a turn-on fluorescent probe: 8-anilino-1-naphthalenesulfonic acid (ANS). The direct detection of proteins via the light-absorbing properties of tryptophan, tyrosine, and phenylalanine has been well established and a widely used diagnostic test involves measuring their overall absorption of 280 nm light, colloquially known as A280. GFP is a protein derived from jellyfish that contains a naturally occurring chromophore that, when excited with 473 nm light, gives off green light that can be used for the direct visualization of tagged proteins and/or cellular mechanisms in cells. ANS is a compound that favors the hydrophobic regions of proteins and transitions from a quenched dark state to one with notable fluorescence; thus, it can readily be used in fluorescent assays for the determination of protein concentration. The importance of this technique comes from its potential as an efficient first-pass testing method for protein detection. In a contemporary GMP testing environment, specific protein detection assays require an understanding of the amount of bulk protein to ensure proper dilutions. The typical first-pass method for this testing involves the use of A280 to understand the baseline of the system. A280 suffers from a limited range of detection, and multiple dilutions steps are typically required prior to this passing this stage. Each dilution step requires man hours for testing, and therefore money. Instead, we propose the use of FIPC to take advantage of their enhanced signal detection range as a first-pass method. Due to the methodology of the testing for FIPC, this testing could also be performed directly at the point of sample collection or in remote working conditions not suitable for UV-vis absorbance spectroscopy, such as on a research vessel in turbid waters or the International Space Station where the vacuum of space prevents the typical fluid motions needed to use a cuvette. Previous work by our group has highlighted the utility of this technique for the detection of environmental contaminants, and illustrated how this technique could be used to facilitate and improve public health outcomes [23,24]. In this study, we examined all three of these mechanisms and how FIPC can be used to develop a robust detection platform for proteins that *does not require* the classical fluorescence detection scheme and the use of a photodetector.

## 2. Materials and Methods

In this study, we utilized metal-nanoparticle films (MNFs) produced by thermal vapor deposition, as outlined in previous work by our group [22]. Copper films were chosen for their relative inertness compared to silver films. These films were produced through affixing a silane-coated glass slide to the deposition area in an Edwards 306 Auto Depositor, directly next to a quartz crystalline deposition sensor. A small piece of pure copper metal was placed into the molybdenum sublimation boat, and a current of 4 A was run through them while under a vacuum of 2E-5 Torr. The metal was sublimated via current-induced heating and the resulting metal gas was deposited on the slides until the desired thickness was registered from the adjacent quartz sensor. The deposition rate was held at a constant 0.1 Å/s to control for the size and distribution of the nanoparticles. The films were allowed to cool before they were electrically and optically characterized to ensure both strong spectral overlap with the fluorophore/protein species and the island-like nature of the particles to ensure that FIPC could be detected. These films were made in the same batch described in [22], and Supplemental Figure S2 details these characterization experiments.

The preparation of the solutions for the A280 analog experiment were straightforward. Bovine Serum Albumin (BSA, Sigma-Aldrich, St. Louis, MO, USA) was dissolved in deionized water to the desired concentrations (200 µg/mL–10 mg/mL) and used as a stock solution for the ANS (Sigma-Aldrich, St. Louis, MO) solutions. The initial ANS stock concentrations were prepared via dilution with deionized water, and the 0.0253 mM ANS stock solutions were spiked with various concentrations of BSA (3.3 µg/mL–666 µg/mL final concentration) in 3 mL of the ANS stock prior to testing. Finally, the GFP from recombinant Aequorea solutions were allowed to thaw from a previously frozen state and then diluted with DI water to the desired concentration for testing (12.5 µg/mL–200 µg/mL). These solutions were tested for their absorption and fluorescence characteristics using a UV-Vis spectrophotometer (Agilent Technologies, Cary 60 UV-Vis, Savage, MD, USA) and spectrofluorometer (Horiba Fluoromax-4P, Kyoto, Japan). ANS fluorescence spectra were obtained from excitation with 473 nm light, while the GFP solutions were excited with 405 nm light.

The FIPC data that we report in this paper were collected following the methodology outlined by our group in previous publications [17,18,19,20,21,22,23]. In short, the MNFs of choice (in this case, 3 nm Cu films) were adhered to the sample stage and metal electrodes (in this case, copper electrodes) were affixed to the surface. The electrodes were connected in series to a picoammeter to detect the current changes in real time during the experiment. A 200 µL volume of the desired solution was then deposited via a micropipette onto the surface of the film, between the two electrodes, in such a position as to allow contact between both electrodes, the solution, and the film. The solutions were then excited from the far-field via a CW laser: for the A280 BSA series, a 266 nm laser was used; a 473 nm laser was used for the ANS testing; and a 405 nm laser was used for the GFP testing. All of these lasers were kept at a constant power of 10 mW, allowed 15 min to warm to full power, and were angled such that they would contact the film/solution at an incidence angle of 45°. This angle is important since an appropriate half wave plate can be used to selectively choose between S and P polarized light for the purposes of examining the fluorophore–metal dipole coupling interactions. These excitation sources were manually gated via a series of optics to allow for the selective excitation of the solution in 1 s intervals, without disrupting the power source of the lasers. The picoammeter in series with the solutions continuously displays the current of the system, while providing a small background bias current of 0.005 v to control for the anode and the cathode of the system. Upon the ungating of the excitation source, the change in the current was noted and processed as one data point. Each data point shown is the average of 30 such individual excitations and current generation cycles, which were derived from the modulus of the current both before and after the excitation of the solution. Supplemental Figure S1 shows the FIPC optical detection scheme employed in these experiments. Other contemporary protein detection methods are detailed in [25,26,27,28,29,30,31,32,33,34].

## 3. Results and Discussion

### 3.1. Direct Detection of BSA Analogous to an A280 Assay

**Figure 1:**
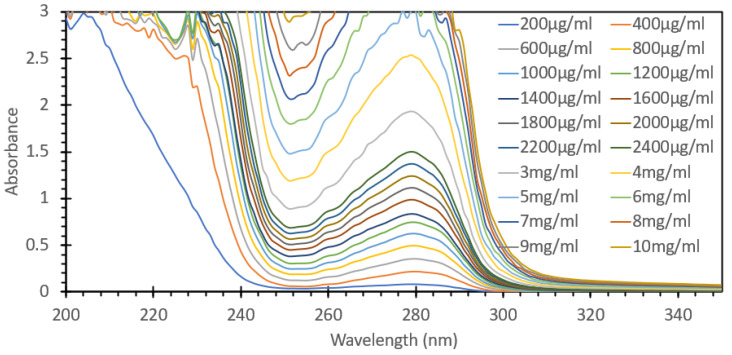
Absorbance spectra of several solutions of BSA, ranging in concentration from 200 µg/mL to 10 mg/mL.

**Figure 2:**
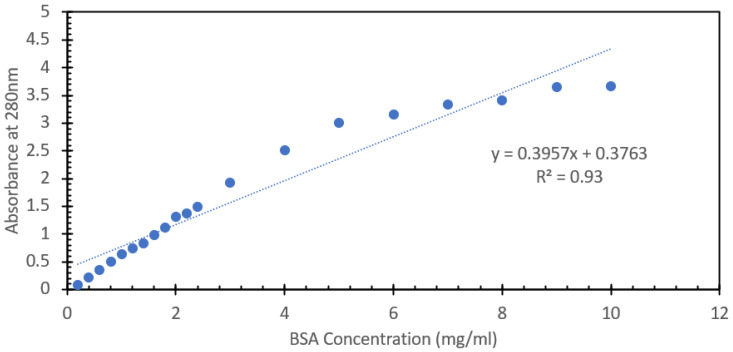
Peak absorbance values at 280 nm of solutions of BSA, ranging in concentration from 200 µg/mL to 10 mg/mL.

**Figure 3:**
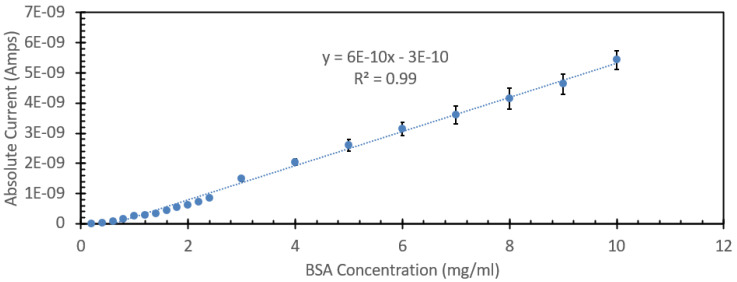
Plasmonic current response of several solutions of BSA, ranging in concentration from 1 mg/mL to 10 mg/mL. Excited at 266 nm and with a power of 100 µW.

One of the most commonly used spectroscopic methods for the analysis and detection of proteins in aqueous solutions is the absorption of the solution at 280 nm. Proteins are comprised of amino acids, and three of them, tyrosine, tryptophan, and phenylalanine, all contain stable ring structure moieties that strongly absorb UV light, with the peak absorption for all three falling between 265 nm and 280 nm. As such, the absorbance of the solution at 280 nm can give a relatively accurate representation of the total concentration of protein in a given solution when paired with a known concentration series that is prepared and analyzed using the Beer–Lambert law. This is a very common first-pass method of detection as there is no requirement for added reagents or time-intensive techniques. However, one of the issues with this approach relates to the concentration range in which it is able to retain its linearity. Many UV-Vis spectrophotometers lose their reliability when the absorbance of a given substance either exceeds one absorbance unit or is less than the limit of detection of the system, typically 0.04 absorbance units. This limitation leads to the assay requiring a series of dilutions and back calculations to determine the nominal concertation of the protein species at hand. For the purposes of this study, we aimed to look at the range and comparability of the FIPC system in detecting these excitable amino acids in a well-studied protein like BSA, and to see how the upper limits of detection could be utilized, relative to the known limitations of UV-Vis spectroscopy. Figure 1 shows the absorbance spectra of concentrations of BSA between 200 µg/mL and 10 mg/mL, limited to the range of between 0 and 3 absorbance units, while Supplemental Figure S3 details all the responses reported by the UV-Vis absorption spectrophotometer. It can be seen that the peak absorbance was at ~280 nm, and that an absorbance value of 1 was reached at the 1600 µg/mL concentration. For the purpose of demonstrating the utility of FIPC, we opted to include the values that exceed the typical limitations of UV-Vis absorption spectroscopy. Figure 2 shows the response of the peak absorbance at 280 nm for the solutions shown in Figure 1, with a linear curve that was fitted to the data. It can be seen that there was a strong linear response up until around 1.5 absorbance units; after that point, we started to see an asymptotic response with increasing concentration, suggesting that for this absorption spectrophotometer machine, the limit of detection for this method would be around 2 mg/mL of BSA. Figure 3 shows the same solutions but with the FIPC system when excited with 266 nm light. A wavelength of 266 nm was chosen for our excitation source as there was still significant absorbance and because the laser was readily available and had been used in prior publications for FIPC. A significant results from this experiment was the linearity that was observed between 200 µg/mL and 2 mg/mL, all the way through to the upper limit of our testing at 10 mg/mL. When these data points were linearized with a standard linear least-squares type fit, they were found to have an R^2^ value of 0.99, indicating that there was a strong linear relationship between BSA concentration and the response beyond the typical limit of detection for this type of test, i.e., the response that would be obtained by using an absorbance spectrophotometer. This finding was of particular interest to us as it reinforced a previous notion of ours that the surface-based responses of FIPC would be able to counteract the concentration-based effects of the inner filter effect and the limit of absorption/transmission when using UV-Vis spectroscopy.

### 3.2. Detection of BSA via Turn-On Fluorescent ANS Probe 

The next method of protein detection we studied with our FIPC approach was using the fluorescent probe ANS. ANS is a ring-structured sulfonic acid with an amine group that is fluorescent, but this fluorescence is rapidly quenched when exposed to water in aqueous solutions. ANS favors hydrophobic pockets inside proteins and is subsequently able to fluoresce, and the presence of this signal is used analytically to understand both the concentration and the conformational changes of a protein. In this experiment, we compared the fluorescence of ANS in several solutions of BSA to the responses generated via FIPC on a Cu nanoparticle substrate when excited with the same wavelength of light.

**Figure 4:**
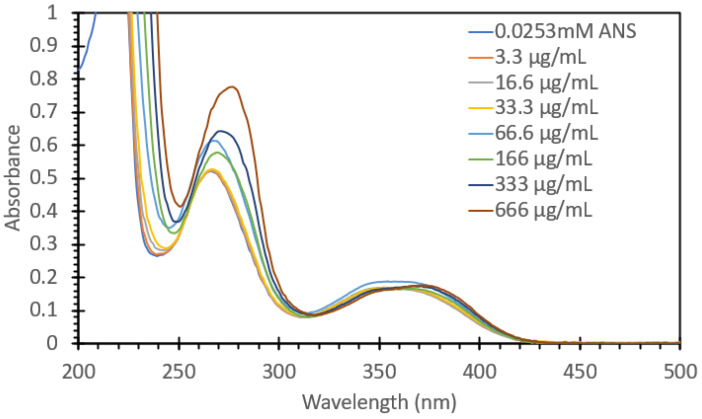
Absorbance spectrum of 0.0253 mM ANS with various concentrations of BSA. Solutions containing BSA are labeled with their BSA concentration.

**Figure 5:**
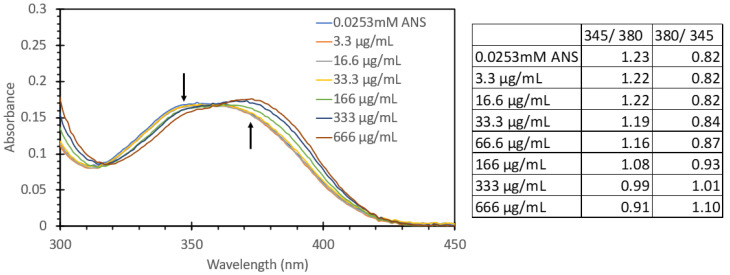
Absorbance spectrum of 0.0253 mM ANS treated with various concentrations of BSA showing the change in absorption at both 345 nm and 380 nm, marked with black arrows, with increasing BSA concentration (**left**). Ratio of peak values at 345 nm and 380 nm (**right**).

**Figure 6:**
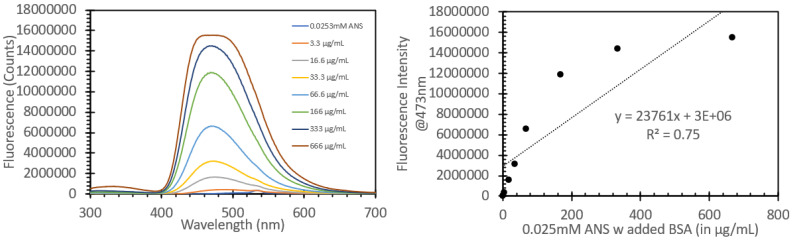
Fluorescence spectra of various solutions of BSA treated with ANS, labeled with BSA concentration, when excited with 473 nm light (**left**) and peak fluorescence values at 473 nm for the same solutions (**right**).

Figure 4 shows the absorbance spectrum for absorbance values between 0 and 1 of 0.0253 mM ANS treated with 100 mL aliquots of solutions with different BSA concentrations (total concentrations were between 3.3 µg/mL and 666 µg/mL); the full range of absorbance values can be found in Supplemental Figure S4. In the 200–300 nm region, a gradual red shift of the observed spectrum was noted, with a general trend of increasing intensity relative to the protein concentration. Figure 5 shows the absorbance spectrum between 300 and 450 nm; it can be readily observed that there was a marked shift in peak absorbance, from a peak at 345 nm for ANS (even with a low amount of protein) to 380 nm when the probe was sitting within the hydrophobic region within the protein. Figure 5 also shows the ratio of the peak absorbance at 345 nm and 380 nm to highlight the shift between the ANS alone and the ANS immobilized with protein to further highlight that there was a marked shift upon the addition of the protein solution with regard to absorbance. These solutions were subsequently tested for their fluorescence responses (Figure 6). The solutions were excited with 473 nm light, i.e., the same wavelength used for FIPC testing. It can be noted that as we approached concentrations of 333 µg/mL and above, we began to see evidence that we had reached the limit of detection for the fluorimeter. The fluorescence peak began to broaden and then ultimately became flat once we hit the limit of detection, with the detector saturating at ~15,000,000 counts. The graph on the right in Figure 6 shows the peak fluorescence values at 473 nm vs. the protein concentration. It can be seen that beyond 166 µg/mL, we began to lose the linear relationship between concentration and peak fluorescence response, indicating that in this particular setup, the range in which this assay would provide useful information for the determination of protein concentration would be between 3.3 µg/mL and 166 µg/mL.

**Figure 7:**
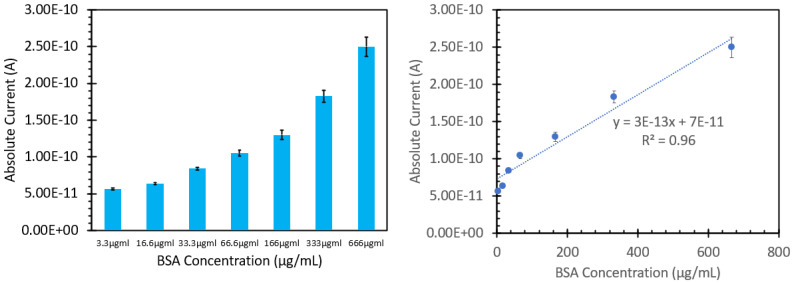
FIPC responses for various solutions of BSA treated with ANS (labeled with BSA concentration) when excited with 266 nm light with a power of 10 mW (**left**). Same responses organized as a standard curve with R2 value and fitted trendline (**right**).

**Figure 8:**
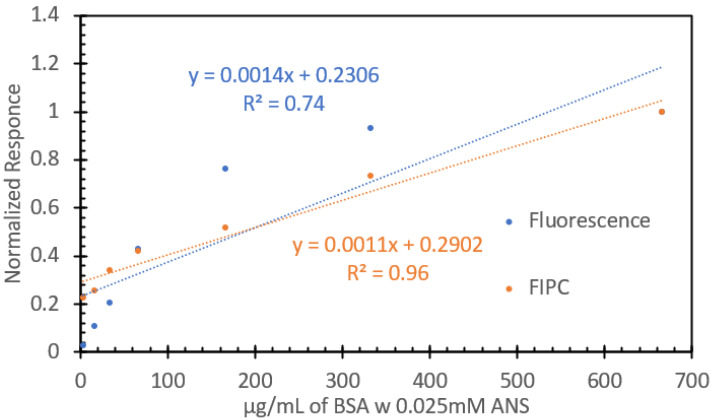
Normalized responses for various concentrations of BSA with ANS added. Peak fluorescence values at 473 nm (blue) and FIPC responses to excitation at 266 nm (orange).

We then studied how the ANS/BSA solutions would respond and operate in our FIPC analysis setup. The solutions were affixed to 3 nm Cu films, attached in series to the current-detecting picoammeter via Cu electrodes, and subsequently excited with 473 nm light at a power of 10 mW, in a series of 30 gated pulses per concentration; the data are shown in Figure 7. The graph on the left in Figure 7 shows the absolute current responses for the solutions; it can be noted that as the concentration of added protein increased, we saw a notable increase in the FIPC response. The right-hand side shows a linearized version of the same data, highlighting the responses as the concentration increased. It can be noted that at higher concentrations, the error bars became larger as the signal increased; however, all the points were found to be statistically different from each other using a standard *t*-test. Unlike the data shown in Figure 6, we saw no indication of an upper limit of detection for these solutions, and we saw similar degrees of linearity throughout the concentration series. Figure 8 compares the linear fits of both the peak fluorescence values and the absolute FIPC values after normalization. The peak fluorescence values at 473 nm are shown in blue, while the absolute FIPC values from are shown in orange. It can be seen that they both exhibit linearity at concentrations from 3.3 µg/mL to 66 µg/mL, but after that, they began to diverge. It can be noted that the fluorescence response was stronger at the lower concentrations, whereas the FIPC response seemed optimal at higher protein–ANS concentrations.

### 3.3. Detection of GFP (Green Fluorescent Protein) via FIPC

The final method of protein detection we examined using the FIPC approach was the detection of a highly fluorescent protein: GFP. The GFP used in this analysis was derived from Aequorea Victoria. The recombinant version we used in this analysis is structurally very similar to those used in genetics and microbiology studies to mark and track the presence of a desired organelle or protein in a live cell. Outside of microbiology and genetics, there are several types of diagnostic assays that involve either the quenching or activation of GFP bound to a target analyte or species. GFP is known for its large *beta*-barrel structure, with its dipole of excitation being directly perpendicular to the relatively large 27 kDa protein. One of our particular interests with this protein is examining how this protein will respond to selective light polarization and excitation due to its large size and relatively large anisotropy compared to smaller chromophores.

**Figure 9:**
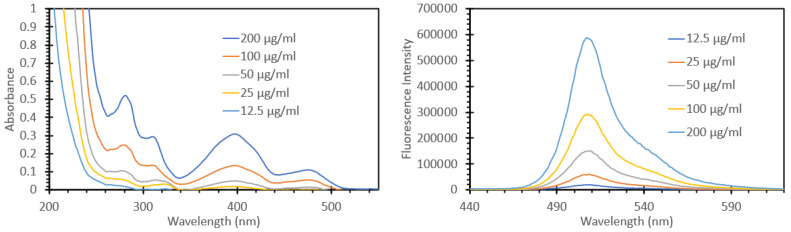
Absorbance spectrum (**left**) and fluorescence spectrum of recombinant Aequorea GFP when excited with 405 nm light (**right**).

After reconstitution according to the manufacturer’s instructions, the GFP was diluted in DI water to the desired concentrations, and the absorbance and fluorescence emissions were measured. Figure 9 shows the spectra obtained from these solutions, with the left-hand side graph showing the absorbance and the right-hand side graph showing the fluorescence when excited with 405 nm light. It can be seen that these spectra were very similar to those reported in the literature, and we can see increasing responses to increasing concentration in both the absorbance and the fluorescence emission spectra, as expected. We also noted a strong overlap between the absorbance and fluorescence emission of the GFP and the properties of our films used for FIPC (the absorption spectra for our 3 nm Cu films can be seen in reference [22]).

**Figure 10:**
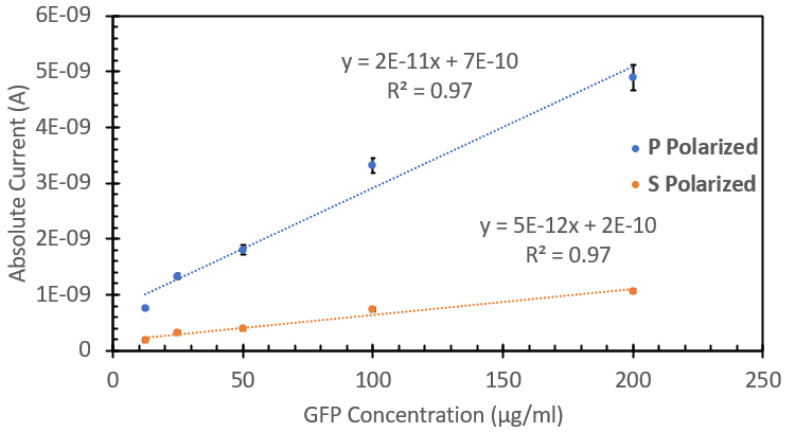
FIPC responses of recombinant Aequorea GFP, with both **S** and **P** polarizations shown, excited with 405 nm light using a power of 10 mW.

Figure 10 shows the FIPC responses of the GFP solutions when excited with both **P** and **S** polarized light. Using an angle of incidence of 45° to the sample surface, the use of a half wave plate allows for selective excitation using either **S** or **P** polarized light, as described in detail elsewhere [20,21,22]. We found that for both orientations, there was an increase in the response with increasing concentration. As expected, the **P** polarized light-induced current was greater in magnitude than that of the **S** polarized light. This effect had been described previously by our group [20,21,22] and is due to the fact that in the **S** configuration, fluorophore-induced dipoles in the metal-nanoparticle film partially cancel out [20,21,22]. It was also noted from this polarized light analysis that the induced current difference between the polarizations was larger than any other values we had observed before for smaller chromophores, with some of the current differences being up to 5x larger. This finding suggests that fluorescent proteins could be used in FIPC polarization-based assays.

## 4. Conclusions

In this study, we studied the use of Fluorophore-Induced Plasmonic Current for the detection of proteins in aqueous solutions. With the advent of modern diagnostic assays, it is imperative to find new techniques that are both rapid and inexpensive. The expanded upper range of detection that was observed for both absorbance and fluorescence measurements for the A280 and ANS assays demonstrate the utility of our FIPC method. Future testing regimens could seek to employ this technique to obtain a robust response without the need for dilutions, while only using ~100 µL of the solution. The direct detection of GFP potentially allows for new microbiological experiments to be performed without the need for an optical examination of the system, which may be harmful to the species being examined. Cell viability assays could potentially be performed in entirely dark environments or without breaking the seal on infectious materials by having the substance be in contact with the FIPC nanoparticle-coated substrate. These findings leave us hopeful for the future expansion of the use of FIPC in diagnostic assay platforms that could potentially be performed in the field at the point of sample collection. Future work for this technology should include the development of specific antibody-based protein detection assays to detect species such as the spike protein of SARS-CoV2. 

## Data Availability

Data are contained within the article and Supplementary Materials.

## Supplementary Materials

The following supporting information can be downloaded at: https://www.mdpi.com/article/10.3390/bios15030150/s1, Figure S1: Schematic of the plasmonic current detection setup; Figure S2: Absorbance spectra of a thermally deposited 3 nm copper film (left) from voltage vs. current sweep experiments (columbic staircase). Increasing voltages were applied to a 3 nm thick thermally evaporated copper film (right); Figure S3: Absorbance spectra of several solutions of BSA, ranging in concentration from 200 µg/mL to 10 mg/mL, with absorbance values up to 10; Figure S4: Absorbance spectrum of 0.0253 mM ANS with various concentrations of BSA. Solutions containing BSA are labeled with their BSA concentrations. Full range of absorbance values up to 10 is shown.

## Abbreviations

MEF—metal-enhanced fluorescence; FIPC—fluorophore-induced plasmonic current; ANS—8-anilino-1-naphthalenesulfonic acid; GFP—green fluorescent protein; BSA—Bovine Serum Albumin.
