# Supplementary material for: Direct Detection and Quantification of Aqueous Proteins via a Fluorescent Probe Through the Use of Fluorophore-Induced Plasmonic Current
Source: Biosensors (Basel). 2025 Feb 27;15(3):150. doi: 10.3390/bios15030150 (PMC11940231; doi:10.3390/bios15030150)
Supplement: Supplementary file 1 [file biosensors-15-00150-s001.zip › biosensors-3477244-supplementary.pdf]

biosensors

MDPI

Supplementary

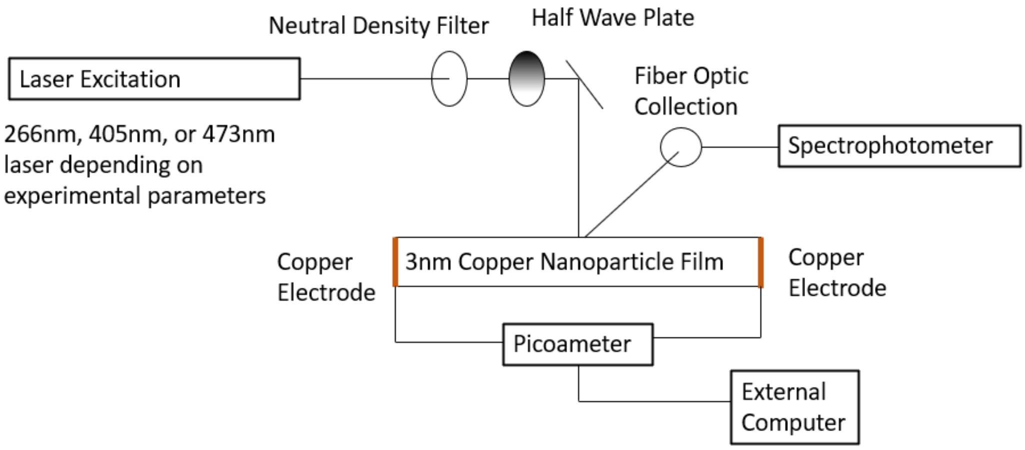

Supplemental Figure S1. Schematic of the plasmonic current detection setup.

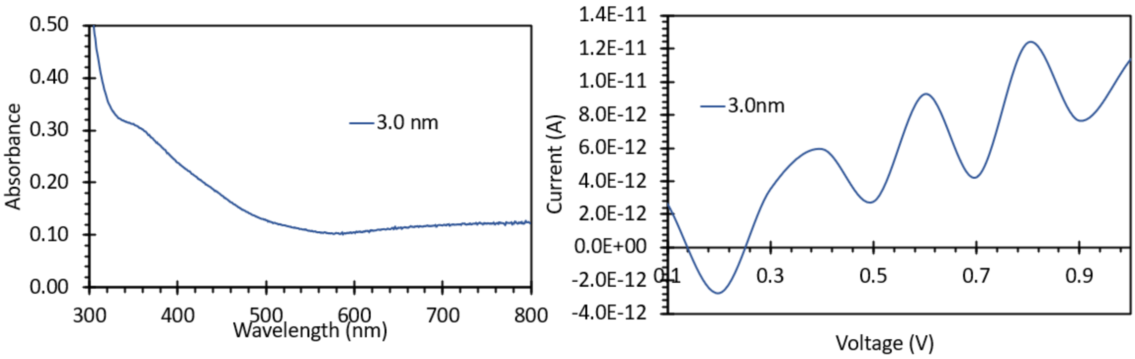

Supplemental Figure S2. Absorbance spectra of a thermally deposited 3nm Copper film (Left), Voltage Vs Current sweep experiments (columbic staircase), for increasing voltages applied to a 3nm thick thermally evaporated Copper film (Right).

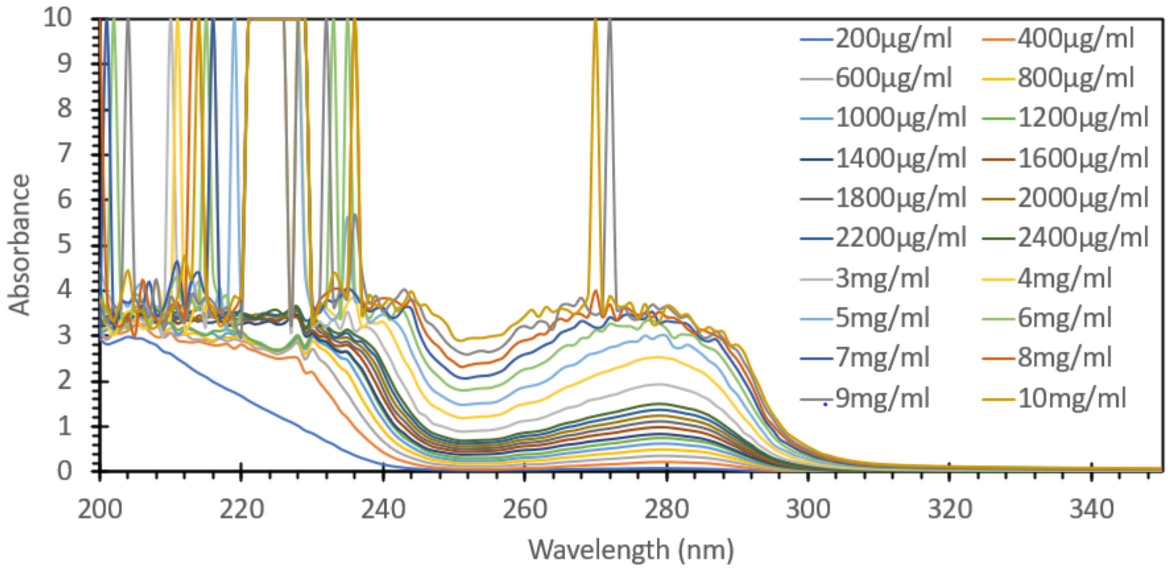

Supplemental Figure S3. Absorbance spectrum of several solutions of BSA, ranging from 200µg/ml to 10mg/ml, with absorbance values up to 10.

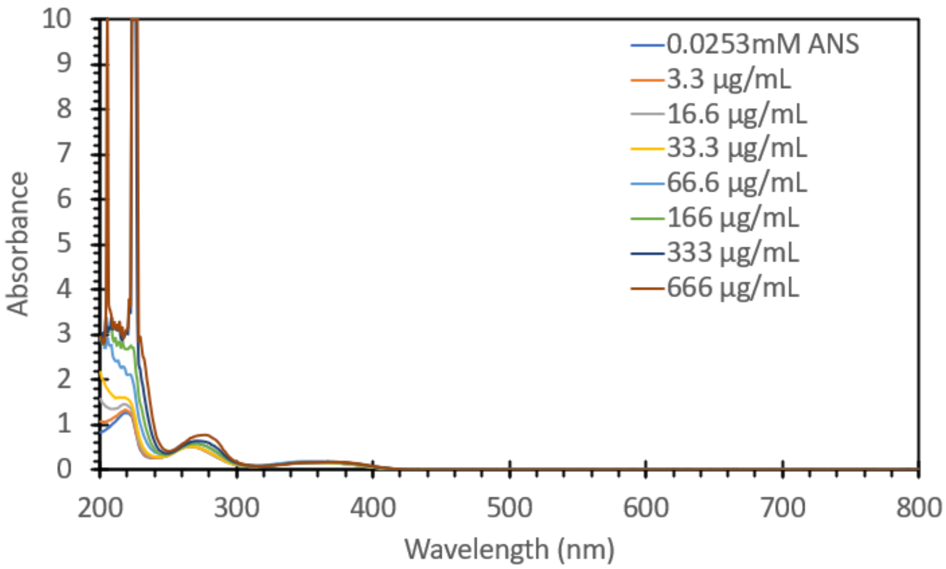

Supplemental Figure S4. Absorbance spectrum of 0.0253mM ANS with and without various concentrations of BSA. Solutions containing BSA labeled by their BSA concentrations, full range of absorbance values up to 10
